# Gait-Based Person Identification Robust to Changes in Appearance

**DOI:** 10.3390/s130607884

**Published:** 2013-06-19

**Authors:** Yumi Iwashita, Koji Uchino, Ryo Kurazume

**Affiliations:** Department of Advanced Information Technology, Kyushu University, 744 Motooka, Nishi-ku, Fukuoka 819-0395, Japan; E-Mails: uchino@irvs.ait.kyushu-u.ac.jp (K.U.); kurazume@ait.kyushu-u.ac.jp (R.K.)

**Keywords:** gait, person identification, affine moment invariants, local features

## Abstract

The identification of a person from gait images is generally sensitive to appearance changes, such as variations of clothes and belongings. One possibility to deal with this problem is to collect possible subjects' appearance changes in a database. However, it is almost impossible to predict all appearance changes in advance. In this paper, we propose a novel method, which allows robustly identifying people in spite of changes in appearance, without using a database of predicted appearance changes. In the proposed method, firstly, the human body image is divided into multiple areas, and features for each area are extracted. Next, a matching weight for each area is estimated based on the similarity between the extracted features and those in the database for standard clothes. Finally, the subject is identified by weighted integration of similarities in all areas. Experiments using the gait database CASIA show the best correct classification rate compared with conventional methods experiments.

## 1. Introduction

Person recognition systems have been used for a wide variety of applications, such as surveillance applications for wide area security operations and service robots that coexist with humans and provide various services in daily life. Gait is one of the biometrics that does not require interaction with subjects and can be performed from a distance. Gait recognition approaches generally fall into two main categories: (1) model-based analysis; and (2) appearance-based analysis. Model-based approaches include parameterization of gait dynamics, such as stride length, cadence, and joint angles [1–4]. Traditionally, these approaches have not reported high performance on common databases, partly due to the self-occlusion caused by legs and arms crossing.

Appearance-based analysis [5,6] uses gait features measured from silhouettes by feature extraction methods, such as gait energy image (GEI) [7], Fourier transforms [8,9], affine moment invariants [10], cubic higher-order local auto-correlation [11], and temporal correlation [12]. Gait features from silhouettes can be separated into static appearance features and dynamic gait features, which reflect the shape of human body and the way how people move during walking, respectively. Katiyar *et al.* propose motion silhouette contour templates and static silhouette templates, which capture the motion and static characteristics of gait [13]. Among several methods to extract gait features, GEI has received the most attention, primarily due to its high performance. GEI improvements have been made and methods based on GEI have been proposed, such as gait flow image (GFI) [14], enhanced gait energy image (EGEI) [15], frame difference energy image (FDEI) [16], and dynamic gait energy image (DGEI) [17]. However, the low contrast between the human body and a complex background is prone to superimposing significant noise levels on silhouette images. To deal with this problem, Kim *et al.* introduced a method to recognize the human body area based on an active shape model [18], and Yu *et al.* proposed a method that reduces the effect of noise on the contour of the human body area [19]. Wang *et al.* proposed a chrono-gait image where gait sequence was encoded to a multichannel image as color information, and showed its robustness to surrounding environment through their experiments [20].

Overall, appearance-based approaches have been used with good results for human identification. Iwama *et al.* built a gait database, which included 4007 people, to show a statistically reliable performance evaluation of gait recognition [21], and they showed that GEI [7] achieved the highest performance among conventional methods. We also showed the robustness of the vision-based gait recognition to the decrease of image resolution [22].

However, since image-based gait recognition is sensitive to appearance changes, such as variations of clothes and belongings, the correct classification rate is reduced in case the subject appearance is different from that in the database. To deal with this problem, several methods to reduce the effect of appearance changes have been proposed [23–27]. Hossain *et al.* [23] introduced a part-based gait identification method. In this method, they predicted the subject's appearance changes in advance, and collected a database that includes these appearance changes. However, it is almost impossible to predict all appearance changes. The correct classification rate would be reduced in case that subject's clothes are not included in the database. Li *et al.* proposed a partitioned weighting gait energy image, which divides a body area into four parts. The person identification is done by a weighted integration of all parts [24]. However, the weight for each individual area needs to be predetermined by the user, which creates the premise for biased results, because of this subjective assessment. Thus the correct classification rate will be reduced in case the subject appearance is different from the user's assumption. Bashir *et al.* [25] introduced the Gait Entropy Image (GEnI) method to select common dynamic areas among the subject's image and images in the database. The features are extracted from selected dynamic areas. Zhang *et al.* proposed an active energy image (AEI) method, which is an average image of active regions estimated by calculating the difference of two adjacent images [26]. Collins *et al.* proposed a shape variation-based frieze pattern representation, which captures motion information by subtracting a silhouette image at a key frame from silhouettes at other times [27]. In these three methods, the correct classification rate is reduced if the subject covers his shape with a big cloth, such as a long coat, due to the following reasons: (i) the dynamic area becomes small, so the discrimination capability of extracted features gets low; (ii) these methods utilize only dynamic features, but not static features that have strong discrimination capability.

In this paper, we propose a person identification method robust to appearance changes. By utilizing both dynamic and static features, the proposed method can prevent a recognition decline, even if subject's appearance is different from that in the database. In the proposed method, the human body image is divided into multiple areas, and features for each area are extracted. In each area, by comparing the features with those in the database, which are constructed from people wearing standard clothes, a matching weight is directly estimated, based on the similarities between the feature of the subject and those in the database. In contrast to [28], the similarity is retrieved automatically based on the diversity of features. Therefore, the proposed method does not need a database with predicted appearance changes. Then, the subject is identified by weighted integration of similarities of all areas. Overall, in comparison with state-of-the-art, the contributions of this paper are:

- The adaptive choice of areas that have high discrimination capability
  – A matching weight at each region can be calculated automatically, although a previous method by Hossain *et al.* [23] also considered it. In addition, the proposed method can reduce the influence of noise on silhouette images, if compared with previous methods [25,26]. This will be further discussed in Section 3.
- Experimental results
  – The proposed method is tested on CASIA-B and CASIA-C datasets. We have provided the performance of the proposed method, as well as the comparison with the state-of-the-art published results [25,26].

Researchers have started using RGBD sensors such as Microsoft Kinect [29–31]. However, due to a ranging limit (around 5 m for Kinect and around 10 m for Swiss Ranger SR4000), sensors should be placed close to the subjects. On the other hand, cameras can be placed far from the subjects, for instance 20–160 m away [22], due to the following reason. In [22], the performance with full resolution images, which were captured by a camera installed 20 m away from subjects, was almost the same with that with low resolution images (12.5% of the resolution along each axis). Thus a gait identification system using cameras has a higher potential when used in large open spaces, if compared with RGBD sensors method.

This paper is organized as follows. Section 2 describes the details of the proposed person identification method. Section 3 describes experiments performed using the CASIA database. Conclusions are presented in Section 4.

### 2. Gait Identification Robust to Changes in Appearance

**Figure 1.**
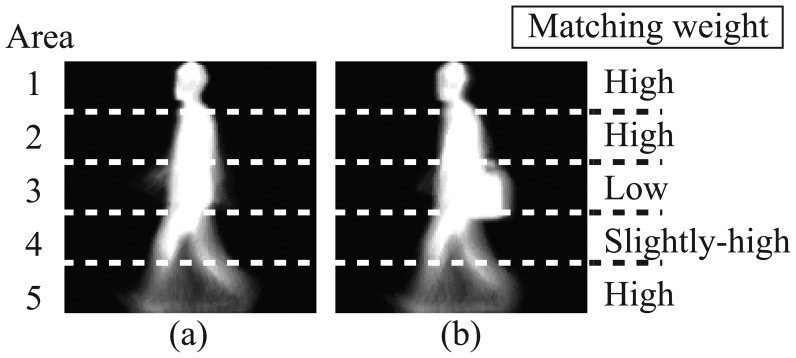
(**a**) An example of average images in the database; (**b**) An example of average images of subjects with a shoulder bag.

In this section, we describe the details of the proposed method. To summarize, the main steps of the identification process are as follows:

Step 1 An average image over a gait cycle is calculated, and then the human body area is divided into multiple areas. Figure 1 shows an example of a human body area divided into 5 areas.
Step 2 Affine moment invariants are extracted at each area as gait features [10]. Database is built from a set of affine moment invariants of multiple people who wear standard clothes without belongings.
Step 3 The average image of the subject person is also divided in the same way as the database, and then gait features are extracted.
Step 4 A matching weight at each area is estimated according to the similarity between the features of the subject and those in the database.
Step 5 The subject is identified by weighted integration of similarities of all areas.

In case that the subject's appearance is different from that in the database as shown in Figure 1, from the above procedure, matching weights of areas with appearance changes are set to low. On the other hand, matching weights of areas with less appearance changes are set to high. Our proposed method does not utilize gait features extracted from areas with low matching weights, which are due to changes of clothes/belongings, but utilizes features from areas with high matching weights. Therefore, the proposed method enables person identification robust to changes in appearance.

### 2.1. Definition of Average Image and Division of Subject's Area

After a silhouette area from a captured image is extracted by a background subtraction method, the human body area is scaled to a uniform height, set to 128 pixels, and the average image *Ī* from images of one gait cycle is defined as follows:

(1) $$\bar{I}(x,y)=\frac{1}{T}\sum_{t=1}^{T}I(x,y,t)$$

where *T* is the number of frames in one gait cycle and *I*(*x*, *y*, *t*) represents the intensity of the pixel (*x*, *y*) at time *t*. Figure 1 shows examples of average images. High intensity values in average images correspond to body parts that move little during a walking cycle, such as head and torso; these areas reflect the human body shape. On the other hand, pixels with low intensity values correspond to body parts that move constantly, such as lower parts of legs and arms. These areas include information about the way how people move during walking. This way, average images include both static and dynamic features.

**Figure 2.**
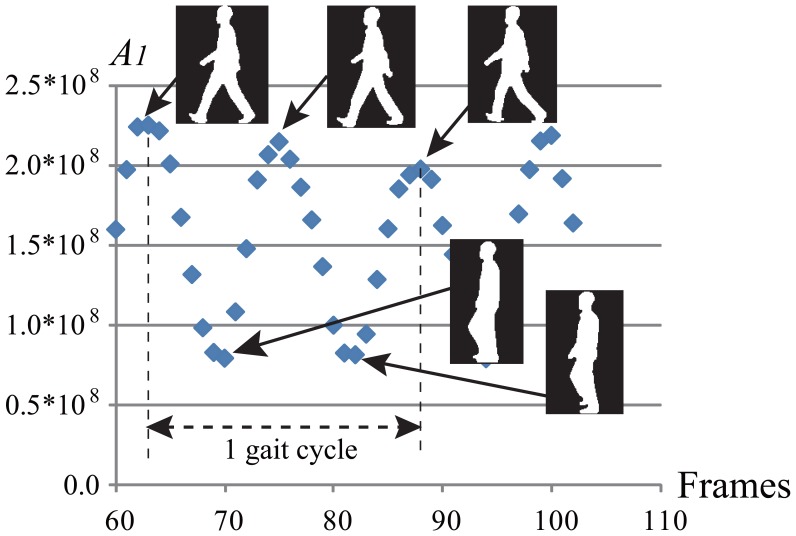
Affine moment invariant *A*_1_ in a gait sequence.

One gait cycle is a fundamental unit to describe the gait during ambulation, which is defined as an interval from the time when the heel of one foot strikes the ground to the time at which the same foot contacts the ground again. Here, we estimate one gait cycle by the following procedure. The first affine moment invariant *A*_1_ explained below is calculated at each frame in a gait sequence as shown in Figure 2. We can see that it is repetitive and frames of local maximal value show a double stance phase. Therefore, we estimate three frames whose values are consecutive local maximums. They determine the images between the first and third frames as those of one gait cycle.

Then, we divide the human body area into *K* equal areas, according to the height. (*K* = 5 in Figure 1).

### 2.2. Affine Moment Invariants

Affine moment invariants are moment-based descriptors, which are invariant under a general affine transform. The derivation of the affine moment invariants originates from the traditional theory of algebraic invariants. The affine moment invariants can be derived in several ways. The most common way is the use of the graph theory. For more details, please refer to [32].

The moments describe shape properties of an object as it appears. For an image, the centralized moment of order (*p* + *q*) of an object *O* is given by

(2) $$\mu_{pq}=\sum \sum_{(x,y)\in O}{\left(x-x_{g}\right)}^{p}{\left(y-y_{g}\right)}^{q}\bar{I}(x,y)$$

Here, *x_g_* and *y_g_* define the center of the object. More specifically, *x_g_* and *y_g_* are calculated from the geometric moments *m_pq_*, given by $x_{g}=\frac{m_{10}}{m_{00}}$ and $y_{g}=\frac{m_{01}}{m_{00}}$, where $m_{pq}=\sum \sum_{(x,y)\in O}x^{p}y^{q}\bar{I}(x,y)$. In our method, the number of affine moment invariants (***A* =** (A_1_, A_2_, …., A_M_)^T^) is *M*. We show six such invariants [32].

(3) $$\begin{array}{lll} {\text{A}}_{1} & = & \frac{1}{\mu_{00}^{4}}(\mu_{20}\mu_{02}-\mu_{11}^{2})\\ A_{2} & = & \frac{1}{\mu_{00}^{10}}(\mu_{30}^{2}\mu_{03}^{2}-6\mu_{30}\mu_{21}\mu_{12}\mu_{03}+4\mu_{30}\mu_{12}^{3}+4\mu_{03}\mu_{21}^{3})\\ & & -3\mu_{21}^{2}\mu_{12}^{2}\\ A_{3} & = & \frac{1}{\mu_{00}^{7}}(\mu_{20}(\mu_{21}\mu_{03}-\mu_{12}^{2})-\mu_{11}(\mu_{30}\mu_{03}-\mu_{21}\mu_{12})\\ & & +\mu_{02}(\mu_{30}\mu_{12}-\mu_{21}^{2}))\\ A_{4} & = & \frac{1}{\mu_{00}^{11}}(\mu_{20}^{3}\mu_{03}^{2}-6\mu_{20}^{2}\mu_{11}\mu_{12}\mu_{03}-6\mu_{20}^{2}\mu_{02}\mu_{21}\mu_{03}\\ & & +9\mu_{20}^{2}\mu_{02}\mu_{12}^{2}+12\mu_{20}\mu_{11}^{2}\mu_{21}\mu_{03}\\ & & +6\mu_{20}\mu_{11}\mu_{02}\mu_{30}\mu_{03}-18\mu_{20}\mu_{11}\mu_{02}\mu_{21}\mu_{12}\\ & & -8\mu_{11}^{3}\mu_{30}\mu_{03}-6\mu_{20}\mu_{02}^{2}\mu_{30}\mu_{12}+9\mu_{20}\mu_{02}^{2}\mu_{21}^{2}\\ & & +12\mu_{11}^{2}\mu_{02}\mu_{30}\mu_{12}-6\mu_{11}\mu_{02}^{2}\mu_{30}\mu_{21}+\mu_{02}^{3}\mu_{30}^{2}\\ A_{5} & = & \frac{1}{\mu_{00}^{6}}(\mu_{40}\mu_{04}-4\mu_{31}\mu_{13}+3\mu_{22}^{2})\\ A_{6} & = & \frac{1}{\mu_{00}^{9}}(\mu_{40}\mu_{04}\mu_{22}+2\mu_{31}\mu_{22}\mu_{13}-\mu_{40}\mu_{13}^{2}-\mu_{04}\mu_{31}^{2})\\ & & -\mu_{22}^{3} \end{array}$$

In case that *M* (the number of affine moment invariants) and *K* (the number of divided areas) get big, high frequency features are extracted. Features in the high frequency domain may include information on noise and low discrimination capability. To reduce these effects, we keep all affine moment invariants and the divided numbers up to certain values. The parameter *M* and *K* are explained in more detail in the experimental section.

### 2.3. Estimation of Matching Weight and Person Identification

In this section, we explain the details of the estimation of matching weight, based on similarities in each area, as well as the procedure of the weighted integration of similarities of all areas.

At first, affine moment invariants in the database and of the subject are whitened at each area. Next, we determine the distance $d_{n,s}^{k}$ between the features of the subject and those of all datasets in the database as follows.

(4) dn,sk=‖AwSUBk−AwDBn,sk‖

where AwSUBk and AwDBn,sk show the whitened affine moment invariants of the subject and those of a person in the database, respectively. The whitening of the affine moment invariants is done as follows; (i) by applying a principal component analysis to calculated affine moment invariants and projecting them to a new features space; and (ii) by normalizing the projected affine features based on their corresponding eigenvalue. *n*, *s*, and *k* are 1 ≤ *n* ≤ *N* (*N* is the number of people in the database), 1 ≤ *s* ≤ *S* (*S* is the number of sequences of each person; one sequence consists of images of one gait cycle), and 1 ≤ *k* ≤ *K* (*K* is the number of divided areas), respectively. ‖ · ‖ means in the Euclidean norm of ·. In the database, there are *N* people and each person has *S* sequences. The distance $d_{n,s}^{k}$ is calculated between the features of the subject and those of each sequence in the database at each area.

Next, at each area we estimate matching weights based on the similarity between the features of the subject and those in the database. We identify people by weighted integration of similarities of all areas. High matching weights are set to the areas with less appearance changes, and low matching weights are set to those with more appearance changes. We adopt the distance $d_{n,s}^{k}$ as a matching weight at each area; short and long distances mean high and low matching weights, respectively.

**Figure 3.**
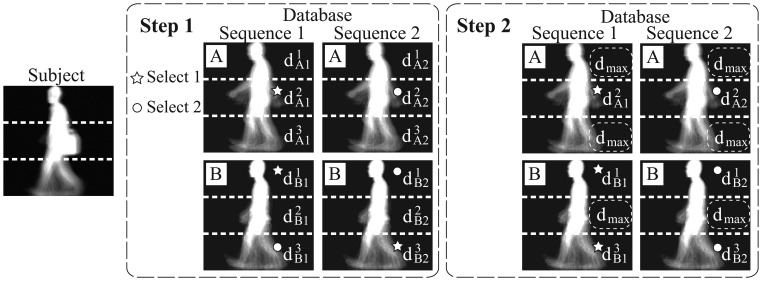
Estimation of matching weights.

The concrete procedure is as follows:

Step 1 At each area *k*, we select sequences from the database if $d_{n,s}^{k}<{\bar{d}}_{\min}^{k}$ shown as the areas with star marks in Figure 3 (select 1), and we consider those selected sequences in the database having high similarities with the subject. Here, the threshold ${\bar{d}}_{\min}^{k}$ is defined as follows.
  (5) $${\bar{d}}_{\min}^{k}=\underset{n}{\min}{\bar{d}}_{n}^{k}$$
  (6) $${\bar{d}}_{n}^{k}=\frac{1}{S}\sum_{s=1}^{S}d_{n,s}^{k}$$
  Moreover, at each area, in case that at least one sequence of a person in the database is selected, we consider that the matching scores of all sequences of the person are also high. This way, even if some of the sequences of a person are not selected, but others are selected, we add these non-selected sequences into selected sequences shown as areas with circle marks (select 2) in Figure 3.
Step 2 We can consider that similarities of non-selected sequences in the database are low, so we redefine the distances of these sequences as a value *d_max_* (*i.e.*, $d_{n,s}^{k}=d_{\text{max}}$ in case $d_{n,s}^{k}\geq {\bar{d}}_{\text{min}}^{k}$. $d_{\text{max}}=max_{n,s,k}d_{n,s}^{k})$ shown as dotted circles in Figure 3. This process allows setting low similarities to the areas of each sequence in the database, which are different from corresponding areas of the subject.
Step 3 The above procedures are applied for all areas.

Finally, the sum of distances for all areas is calculated by $D_{n,s}=\sum_{k=1}^{K}d_{n,s}^{k}$, and the subject is identified by the k-nearest neighbor method. In the experiment, the number *k* of the classifier is 1.

### 2.4. Characteristics of the Proposed Method

The proposed method does not require a database, like the Hossain's method [23], which collects predicted appearance changes, but estimates matching weights directly from features in the database for standard clothes and those of the subject with the appearance change. This way, the proposed method allows identifying people with an unknown appearance change. Moreover, the proposed method utilizes features from not only dynamic areas but also static ones, like head and body. Therefore, it is robust to changes in appearance compared with conventional methods [25,26] that utilize only dynamic features.

## 3. Experiments

This section shows the results of the person identification experiments using the CASIA database (Dataset B and C) [33].

The CASIA-B and CASIA-C datasets comprise 124 subjects' gait sequences collected indoor, and 153 subjects' sequences collected outdoor, respectively. Each gait sequence in the CASIA-B has 11 different view directions, from 0 to 180 degrees between each two nearest view directions. In our experiments, we used the sequences collected at a 90 degree view. The CASIA-C was collected by an infrared camera. For more details, please refer to [33].

**Figure 4.**
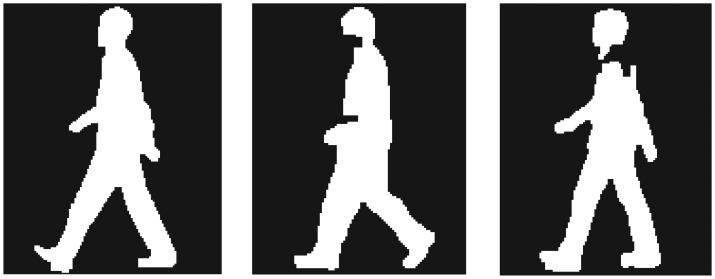
Examples of silhouette images of the CASIA-B dataset.

**Figure 5.**
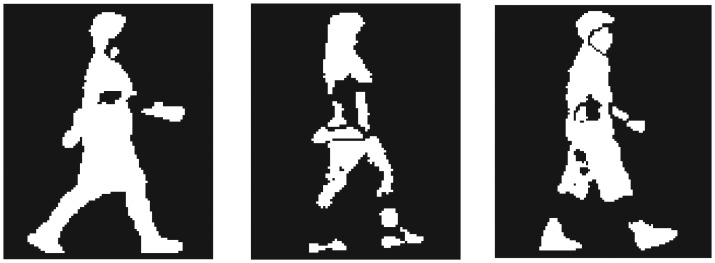
Examples of silhouette images of the CASIA-C dataset.

Figures 4 and 5 show examples of silhouette images from both datasets. Both datasets contain noise and deficit on silhouette images; especially silhouette images in the CASIA-C dataset are of much worse quality. Noise and deficit on silhouette images change the subject appearance and reduce the correct classification rate (CCR). Thus, in the first experiment, we applied the proposed method to walking sequences without appearance changes (hereafter called “standard walking sequences”) from both datasets. In the second experiment, to evaluate the robustness of the proposed method to appearance changes due to variations of clothes and belongings, we applied the proposed method to CASIA-B dataset, which includes carrying-bag and clothes changing sequences.

### 3.1. Person Identification Robust to Noise and Deficit in Silhouette Images

In the first experiments we applied the proposed method to the CASIA-B and CASIA-C datasets. In the experiments we utilized standard walking sequences to check the robustness of the proposed method to noise and deficit. In the CASIA-B dataset for each subject, there are 6 standard walking sequences, and in the CASIA-C dataset there are 4 sequences for each subject.

We compared the proposed method with the conventional methods [25,26], which showed the highest performance among the conventional methods applied to the CASIA database. In [25] the first four sequences of each subject in CASIA-B dataset were used for training datasets, and in [26] three sequences were used (*i.e.*, 2-fold cross validation). In case of the CASIA-C dataset, [25] did not evaluate the method, but [26] did with 4-fold cross validation. This way, we evaluated the proposed method in the same way like [26].

#### 3.1.1. Person Identification with CASIA-B

In this experiment, we applied the proposed method to the CASIA-B dataset. We calculated CCRs in the same way like [26], which implies that the six sequences of each subject were divided into two sets and the method was tested by a 2-fold cross validation method (124 × 3 sequences were used for training and the rest were used for testing).

Here, the CCR was calculated by dividing the number of test datasets, which were classified correctly, by that of all test datasets.

**Figure 6.**
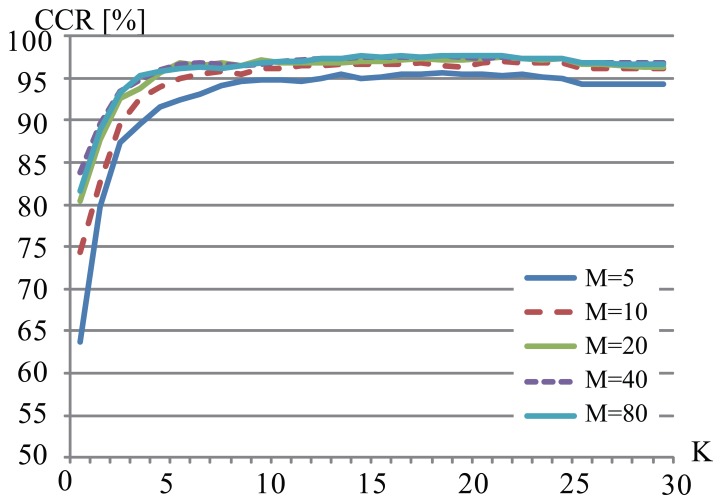
Correct classification rates by the proposed method (CASIA-B).

We changed the parameter *K* from 1 to 30 and the total number of *M* of affine moment invariants from 1 to 80. We tested all combinations of *K* and *M*. Figure 6 shows examples of CCRs (*M* = 5, 10, 20, 40, and 80) with respect to the change of *K*. From this figure, it is clear that the CCR increased with the parameters *K* and *M*. In case that *K* = 17 and *M* = 45 in the CASIA-B dataset, the proposed method showed the highest performance of 97.7%.

**Figure 7.**
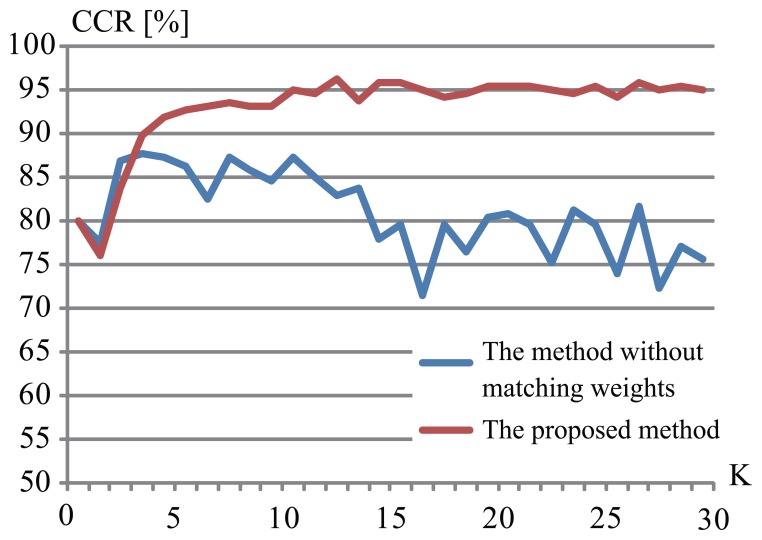
Correct classification rates by the proposed method and the method without matching weights.

To verify the effectiveness of matching weights that we introduced in the proposed method, we did experiments without controlling the matching weights [10] (hereafter called “a method without matching weights”), which means that we did not redefine distances. In this experiment, we set the parameter *M* as 1, and we changed the number of the parameter of *K*. Figure 7 shows the results of the proposed method and the method without matching weights, with respect to the change of the *K* parameter. These CCRs of the method without matching weights are worse than the CCRs of the proposed method. This way, we could verify the effectiveness of controlling matching weights. One of the reasons that the CCR of the method without matching weights was worse is because, as we mentioned before, most of silhouette images publicly available in the CASIA database contain noise and deficit as shown in Figure 4, and the method without matching weights used all areas, even if the similarities of some of them were low. On the other hand, the proposed method allows selecting parts whose similarities were high.

#### 3.1.2. Person Identification with CASIA-C

**Figure 8.**
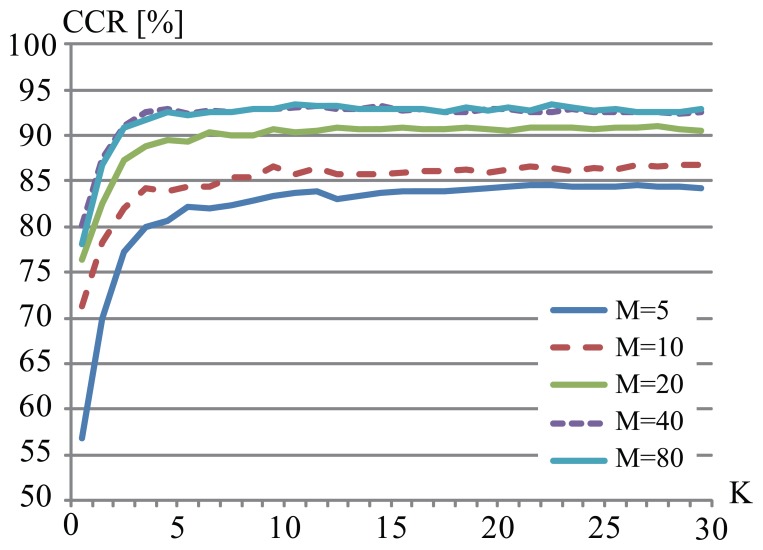
Correct classification rates by the proposed method (CASIA-C).

In this experiment, we applied the proposed method to the CASIA-C dataset. Four sequences for each subject are divided into two sets and the method was tested through a 4-fold cross validation (153 × 3 sequences were used for training and the rest were used for testing). Figure 8 shows examples of CCRs of the CASIA-C dataset. In case that *K* = 7 and *M* = 65 in the CASIA-C dataset, the proposed method showed the highest performance 94.0%. Although silhouette images in the CASIA-C dataset are of worse quality than those in the CASIA-B dataset, the proposed method could identify people with high performance.

#### 3.1.3. Comparison with Conventional Methods

In this experiment we compared the proposed method with conventional methods [25,26]. Table 1 shows results of the CASIA-B and CASIA-C for the proposed method and the conventional methods [25,26]. The CCR of the CASIA-B for the proposed method was almost the same with those for the conventional methods. In case of the CASIA-C, the proposed method outperformed the conventional method [26]. Note that [25] did not evaluate their method with the CASIA-C dataset.

### 3.2. Person Identification Robust to Appearance Changes

**Figure 9.**
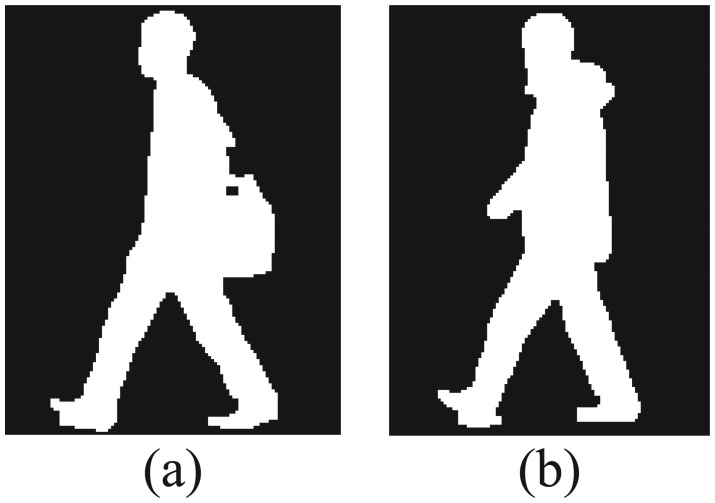
Examples of silhouette images: (**a**) CASIA-B-BG and (**b**) CASIA-B-CL.

In the second experiment, to evaluate the robustness of the proposed method to appearance changes due to variations of clothes and belongings, we applied the proposed method to the CASIA-B dataset, which includes 2 carrying-bag sequences (CASIA-B-BG), and 2 changing clothes sequences (CASIA-B-CL). Figure 9(a,b) shows examples of silhouette images of CASIA-B-BG and CASIA-B-CL, respectively. In the following experiments, we used *K* = 17 and *M* = 45, which showed the highest performance in Section 3.1.1. We compared the proposed method with conventional methods [25,26]. To evaluate the performance, we calculated CCRs in the same way like [26], which implies that the six standard sequences of each subject were divided into two training datasets (*i.e.*, the first 3 and last 3 sequences of each subject were used for each training), and two carrying-bag sequences for CASIA-B-BG and two changing clothes sequences for CASIA-B-CL were used for testing, respectively.

#### 3.2.1. Person Identification with CASIA-B-BG

**Figure 10.**
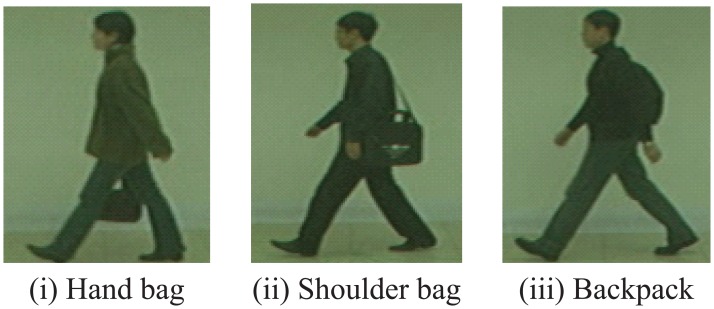
Example images of each category (CASIA-B-BG).

In this experiment, we used CASIA-B-BG as the test datasets. Here, the sequences in CASIA-B-BG can be separated into 4 categories: (i) carrying a handbag (42 sequences); (ii) carrying a shoulder bag (171 sequences); (iii) carrying a backpack (30 sequences); and (iv) others (3 sequences). The category “others” includes sequences in which the subject walked unstably. Figure 10 shows example of each category. The CCR for the proposed method was 91.9%. To verify the effectiveness of matching weights, we did experiments with the method without matching weights. The CCR for the method without matching weights was 20.2%. Table 2 also shows CCR of each category.

**Figure 11.**
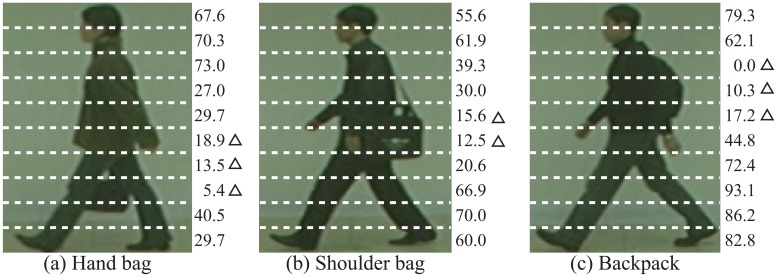
Rate of assigned high matching weight in each area (*K* = 10) [%]. Triangles show areas with appearance changes.

To show that the proposed method adaptively chose areas that had high discrimination capability, at each area we calculated a ratio, which was defined with the subjects classified correctly. At each c. Figure 11 shows examples of the ratios for each category, in case of *K*=10. From these results, we can see that areas without appearance changes have high ratios. On the other hand, areas with appearance changes, such as hand bag area, shoulder bag area, and backpack area, have less ratios.

#### 3.2.2. Person Identification with CASIA-B-CL

**Figure 12.**
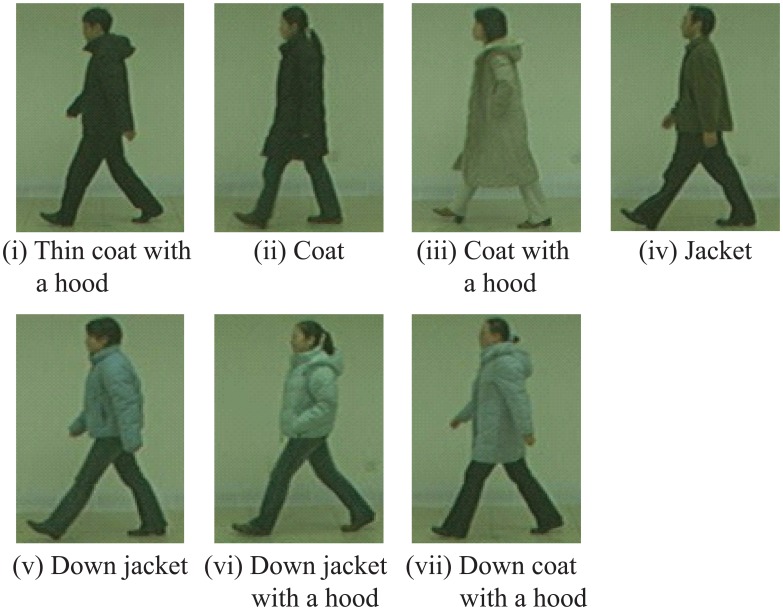
Example images of each category (CASIA-B-CL).

Next, we used CASIA-B-CL as the test datasets. Here, the sequences in CASIA-B-CL can be separated into 7 categories: (i) thin coat with a hood (30 sequences); (ii) coat (24 sequences); (iii) coat with a hood (16 sequences); (vi) jacket (70 sequences); (v) down jacket (62 sequences); (vi) down jacket with a hood (28 sequences); and (vii) down coat with a hood (16 sequences). Figure 12 shows examples of each category. The CCR for the proposed method was 78.0% and that for the method without matching weights was 22.4%. as shown in Table 3. Table 3 also shows CCR of each category.

**Figure 13.**
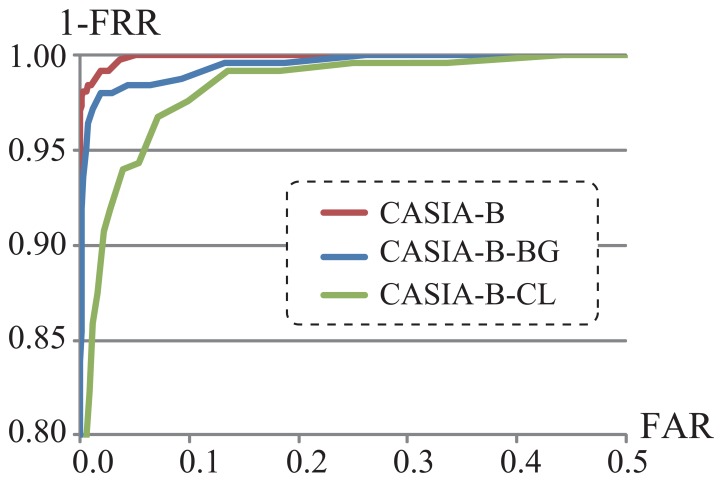
ROC curves by the proposed method (CASIA-B, CASIA-B-BG, CASIA-B-CL).

We evaluated the performance of the proposed method in terms of true positive rates and false positive rates. More specifically, we plotted a Receiver Operating Characteristic (ROC) curve of each dataset CASIA-B, CASIA-B-BG, and CASIA-B-CL as shown in Figure 13, which describes how true positive rate and false positive rate change as the acceptance threshold changes. The threshold was defined by the total number of areas with high matching weights in each person.

#### 3.2.3. Comparison of the Proposed Method with Conventional Methods

We compared the proposed method with conventional methods [25,26]. Table 4 shows the results of CASIA-B-BG and CASIA-B-CL for the proposed method and the conventional methods [25,26]. From these results, it became clear that the proposed method outperformed the conventional methods. In particular, in the case of CASIA-B-CL, some of the subjects covered their body with big clothes. In this case the dynamic area is reduced. This is why the CCRs for conventional methods [25,26] decreased. On the other hand, since the proposed method utilized both dynamic and static features, the proposed method outperformed the conventional methods.

## 4. Conclusions and Future Work

We proposed in this paper a person identification method robust to changes in appearance. In this method, we divided the human body area into multiple areas, and then affine moment invariants were extracted at each area as gait features. In each area, a matching weight was estimated based on the similarity between the features of the subject and those in the database. Then, the subject was identified by weighted integration of similarities in all areas. We carried out experiments with the database CASIA, and showed the robustness of the proposed method compared with conventional methods against appearance changes, especially clothing variety.

In this research we focused on the appearance changes due to variations of clothing and belongings. There are other potential factors that may influence the performance of the gait identification, such as different walking direction, walking speed, *etc.* The specific immediate objective is to develop improved methods that offer robustness to appearance changes due to walking direction changes.

We proposed robust methods to appearance changes in [34,35]. These methods are based on a 4D gait database consisting of multiple 3D shape models of walking people and adaptive virtual image synthesis. Combination of the proposed method with these methods will produce a method that is robust to appearance changes due to both walking direction changes and variations of clothes and belongings.

Future work will also address the second factor, which is the walking speed change. Although the speed change may alter the way people walk, it may have less influence on the moment when a person crosses his legs during walking. Thus the future work will include developing a method that utilizes gait features that are less influenced by walking speed changes.

## Figures and Tables

**Table 1.** Correct classification rates with CASIA-B and CASIA-C by the proposed method and the conventional methods [%].

	**The proposed method**	**Conventional Method I [25]**	**Conventional Method II [26]**
CASIA-B	97.7	100.0	98.4
CASIA-C	**94.0**	N/A	88.9

**Table 2.** Correct classification rates with CASIA-B-BG by the proposed method and the method without matching weights [%].

	**The Proposed Method**	**The Method without Matching Weights**
Total	91.9	20.2
(i) handbag	89.3	8.3
(ii) shoulder bag	92.2	19.9
(iii) backpack	95.0	40.0
(iv) others	83.3	0.0

**Table 3.** Correct classification rate with CASIA-B-CL by the proposed method and the method without matching weights [%].

	**The Proposed Method**	**The Method without Matching Weights**
Total	78.0	22.4
(i) thin coat with a hood	70.3	18.8
(ii) coat	62.5	6.3
(iii) coat with a hood	53.1	6.3
(vi) jacket	85.7	31.4
(v) down jacket	83.9	25.8
(vi) jacket with a hood	78.6	32.1
(vii) down coat with a hood	84.4	0.0

**Table 4.** Comparison of the proposed method with the conventional methods [25,26][%].

	**The Proposed Method**	**Conventional Method I [25]**	**Conventional Method II [26]**
CASIA-B-BG	**91.9**	78.3	91.9
CASIA-B-CL	**78.0**	44.0	72.2

## References

[1] Bouchrika I., Nixon M. People Detection and Recognition Using Gait for Automated Visual Surveillance.

[2] Cunado D., Nixon M., Carter J. (2003). Automatic extraction and description of human gait models for recognition purposes. Comput. Vis. Image Underst..

[3] Yam C., Nixon M., Carter J. (2004). Automated person recognition by walking and running via model-based approaches. Pattern Recognit..

[4] Tafazzoli F., Safabakhsh R. (2010). Model-based human gait recognition using leg and arm movements. Eng. Appl. Articial Intell..

[5] BenAbdelkader C., Cutler R., Nanda H., Davis L. EigenGait: Motion-based Recognition of People Using Image Self-similarity.

[6] Liu Y., Collins R., Tsin Y. Gait Sequence Analysis Using Frieze Patterns.

[7] Han J., Bhanu B. (2006). Individual recognition using gait energy image. IEEE Trans. Pattern Anal. Mach. Intell..

[8] Acquah J., Nixon M., Carter J. (2003). Automatic gait recognition by symmetry analysis. Pattern Recognit. Lett..

[9] Sugiura K., Makihara Y., Yagi Y. Gait Identification Based on Multi-view Observations Using Omnidirectional Camera.

[10] Iwashita Y., Kurazume R. Person Identification from Human Walking Sequences Using Affine Moment Invariants.

[11] Kobayashi T., Otsu N. Action and Simultaneous Multiple-Person Identification Using Cubic Higher-Order Local Auto-Correlation.

[12] Sarkar S., Phillips P., Liu Z., Vega I., Grother P., Bowyer K. (2005). The humanID gait challenge problem: Data sets, performance, and analysis. IEEE Trans. Pattern Anal. Mach. Intell..

[13] Katiyar N., Pathak V., Katiyar R. (2010). Human gait recognition by using motion silhouette contour templates and static silhouette templates. VSRD-TNTJ.

[14] Lam T., Cheung K., Liu J. (2011). Gait flow image: A silhouette-based gait representation for human identification. Pattern Recognit..

[15] Lin C., Wang K. A Behavior Classification Based on Enhanced Gait Energy Image.

[16] Chen C., Liang J., Zhao H., Hu H., Tian J. (2009). Frame difference energy image for gait recognition with incomplete silhouettes. Pattern Recognit. Lett..

[17] Zhang E., Ma H., Lu J., Chen Y. Gait Recognition Using Dynamic Gait Energy and PCA+LPP Method.

[18] Kim D., Paik J. (2010). Gait recognition using active shape model and motion prediction. IET Comput. Vision.

[19] Yu S., Tan D., Huang K., Tan T. Reducing the Effect of Noise on Human Contour in Gait Recognition.

[20] Wang C., Zhang J., Wang L., Pu J., Yuan X. (2012). Human identification using temporal information preserving gait template. IEEE Trans. Pattern Anal. Mach. Intell..

[21] Iwama H., Okumura M., Makihara Y., Yagi Y. (2012). The OU-ISIR gait database comprising the large population dataset and performance evaluation of gait recognition. IEEE Trans. Inf. Forensics Secur..

[22] Iwashita Y., Stoica A., Kurazume R. (2012). Gait identification using shadow biometrics. Pattern Recognit. Lett..

[23] Hossain M.D., Makihara Y., Wang J., Yagi Y. (2010). Clothing-invariant gait identification using part-based clothing categorization and adaptive weight control. Pattern Recognit..

[24] Li X., Wang D., Chen Y. (2013). Gait recognition based on partitioned weighting gait energy image. Lecture Notes Comput. Sci..

[25] Bashir K., Xiang T., Gong S. (2010). Gait recognition without subject cooperation. Pattern Recognit. Lett..

[26] Zhang E., Zhao Y., Xiong W. (2010). Active energy image plus 2DLPP for gait recognition. Signal Process..

[27] Lee S., Liu Y., Collins R. (2007). Shape variation-based frieze pattern for robust gait recognition. Comput. Vision Pattern Recognit..

[28] Iwashita Y., Uchino K., Kurazume R. (2011). Person identification robust to changes in appearance (in Japanese). IEICE Tech. Rep..

[29] Milovanovic M., Minovic M., Starcevic D. (2012). New gait recognition method using Kinect stick figure and CBIR. Telecommun. Forum (TELFOR).

[30] Preis J., Kessel M., Werner M., Linnhoff-Popien C. Gait Recognition with Kinect.

[31] Munsell B., Temlyakov A., Qu C., Wang S. Person Identification Using Full-body Motion and Anthropometric Biometrics from Kinect Videos.

[32] Flusser J., Suk T., Zitova B. (2009). Moments and Moment Invariants in Pattern Recognition.

[33] CASIA Gait Database. http://www.sinobiometrics.com.

[34] Iwashita Y., Baba R., Ogawara K., Kurazume R. Person Identification from Spatio-temporal 3D Gait.

[35] Iwashita Y., Baba R., Ogawara K., Kurazume R. Method for Gait-based Biometric Identification Robust to Changes in Observation Angle.

